# Editorial: Revolutionizing sports science: biomechanical models, wearable tech, and AI

**DOI:** 10.3389/fspor.2026.1849247

**Published:** 2026-04-27

**Authors:** Guoxin Zhang, Zixiang Gao, Yinghu Peng, Yang Song, Lei Zhao

**Affiliations:** 1 Medical Engineering & Engineering Medicine Innovation Center, Hangzhou International Innovation Institute, Beihang University, Hangzhou, Zhejiang, China; 2 Department of Biomedical Engineering, Faculty of Engineering, The Hong Kong Polytechnic University, Hong Kong, Hong Kong SAR, China; 3 Human Performance Laboratory, Faculty of Kinesiology, University of Calgary, Calgary, AB, Canada; 4 Guangdong-Hong Kong-Macao Joint Laboratory of Human-Machine Intelligence-Synergy Systems, Shenzhen Institutes of Advanced Technology, Chinese Academy of Sciences, Shenzhen, Guangdong, China; 5 Faculty of Sports Science, Ningbo University, Ningbo, Zhejiang, China; 6 Research Academy of Grand Health, Ningbo University, Ningbo, Zhejiang, China; 7 School of Mechanical and Electronic Engineering, Shandong Jianzhu University, Jinan, Shandong, China

**Keywords:** artificial intelligence, human movement analysis, machine learning, sports biomechanics, wearable sensors

The landscape of sports science is undergoing a profound transformation, driven by the convergence of high-fidelity biomechanical modeling, ubiquitous wearable technology, and the predictive power of Artificial Intelligence (AI). This Research Topic, “Revolutionizing sports science: Biomechanical models, wearable tech, and AI,” curates a collection of 20 innovative studies that illustrate how these technologies are transitioning from highly controlled laboratory environments into real-world athletic, clinical, and rehabilitative settings. By integrating hardware innovation with sophisticated data-driven frameworks, these works collectively aim to enhance human performance, mitigate injury risks, and personalize the user experience in sports and medicine.

1. Wearable Technology and Material Innovation

A significant portion of this topic explores how the physical interface between technology and the human body can be optimized. Footwear and apparel are no longer passive equipment but active components of the biomechanical chain. For instance, Effects of a wrapping closure lacing system on wearing comfort, lock-in stability, and lower-limb muscle demand during prolonged running demonstrates how dial-based lacing systems improve marathoners' comfort and reduce muscular demand compared to conventional laces. The shift toward “invisible” and comfortable technology is further highlighted in Fully textile passive wireless sensing for human movement monitoring with multiple sensors, which introduces a multi-sensor textile system that monitors movement without rigid electronics.

The interaction between apparel and the body is quantified in A novel anthropometric method to accurately evaluate tissue deformation, providing designers with a tool to measure how compression garments affect soft tissue. Beyond apparel, real-time feedback systems are evolving; Effects of a visual-feedback LED pacing system in middle-distance pool freestyle swimming shows that underwater LED pacing significantly optimizes training load management by stabilizing heart rate and reducing lactate accumulation. Finally, the holistic utility of wearables in tracking long-term environmental and lifestyle impacts is demonstrated in A case study on the impact of Ramadan on biomechanical and physiological markers in a female collegiate student-athlete.

2. AI-Driven Analytics and Computer Vision

The decoding of athletic movement is being revolutionized through AI and computer vision, often using accessible hardware like smartphones. Development of a MediaPipe-based framework for biomechanical quantification of table tennis forehand strokes provides a lightweight solution for identifying kinematic parameters contributing to performance. In the realm of elder care, Gait stability prediction through synthetic time-series and vision-based data utilizes synthetic data generation to improve the prediction of gait stability, overcoming data scarcity in vulnerable populations.

Technical maturity in AI is also addressed through foundational improvements. Self-supervised learning enhances accuracy and data efficiency in lower-limb joint moment estimation from gait kinematics demonstrates how pre-training on unlabeled data reduces the need for expensive labeled datasets. To ensure these models are ready for clinical use, Explainable artificial intelligence for gait analysis: advances, pitfalls, and challenges—a systematic review emphasizes the necessity of interpretability. Furthermore, data-driven personalization is explored in A machine learning approach for saddle height classification in cycling, offering an objective tool for bike fitting based on individual dynamic variations.

3. Rehabilitation and Assistive Technologies

Technological intervention is proving transformative for recovery and injury prevention. An adaptive hand exoskeleton rehabilitation training system integrating virtual reality and an AI-based assessment engine exemplifies a holistic approach to neurorehabilitation. For training safety, Development of a training-oriented wearable knee joint exoskeleton for forming a scientific force application pattern in squat tasks seeks to actively guide users toward safer, hip-dominant movement patterns.

Surgical and orthopedic innovations also feature prominently. Biomechanical and clinical evaluation of 3D-printed integrated tibial prosthesis for reconstructing AORI type Ⅲ tibial plateau defects showcases the superior stability of personalized implants. Similarly, Biomechanical design of titanium-PEEK combined fusion cage based on PLIF surgical model uses machine learning to optimize the structural parameters of spinal implants, reducing stress shielding and enhancing postoperative stability.

4. Biomechanical Insights into Performance and Health

Understanding the underlying mechanisms of movement remains central to sports science. Kinematic and muscle synergy patterns of the lower limbs during jump-landing with side-cutting in individuals with functional ankle instability reveals how injury leads to neuromuscular reorganization. Fatigue, a critical factor in performance, is examined in Biomechanical differences in lower limb movements during lifting tasks before and after fatigue, identifying high-load thresholds where fatigue increases musculoskeletal risk.

The study of stability is extended in Asymmetry of muscle co-activation during the two-armed kettlebell swing: insights into neuromuscular stability, which clarifies the role of co-activation in performance and injury risk. Beyond physical mechanics, Evaluation of the effects of the body on athletes' emotions and motivational behaviors from the perspective of big data public health applies optimization algorithms to recognize emotional states from physiological signals, emphasizing the mind-body connection in high-performance environments.

5. Emerging Trends and Digital Engagement

The research topic also captures how technology influences the broader ecosystem of sport. Enhancing adaptations to peripheral distractors during basketball throwing in real world through virtual reality application investigates using VR to train athletes to filter out sensory noise. Finally, bridging the gap between performance and the sports industry, Distinct drivers of stadium attendance and online streaming: evidence from the Chinese women's super league underscores the growing role of data-driven approaches in advancing audience research and fan engagement in the digital era.

## Conclusion

The 20 articles in this Research Topic represent a snapshot of a future where sports science is increasingly personalized and data-driven. From the molecular-level optimization of 3D-printed implants to the macroscopic analysis of league-wide viewership, the fusion of biomechanics, wearables, and AI is not just revolutionizing how we train and recover, but also how we understand the complex limits of human potential.

